# Disability-Inclusive Diabetes Self-management Telehealth Program: Protocol for a Pilot and Feasibility Study

**DOI:** 10.2196/31689

**Published:** 2021-09-10

**Authors:** Eric Evans, Ayse Zengul, Allyson Hall, Haiyan Qu, Amanda Willig, Andrea Cherrington, Mohanraj Thirumalai

**Affiliations:** 1 Department of Health Services Administration University of Alabama at Birmingham Birmingham, AL United States; 2 Department of Nutrition Sciences University of Alabama at Birmingham Birmingham, AL United States; 3 Department of Medicine University of Alabama at Birmingham Birmingham, AL United States

**Keywords:** telehealth, health coaching, artificial intelligence, diabetes mellitus, mobile phone

## Abstract

**Background:**

Individuals with disabilities and type 2 diabetes require self-management programs that are accessible, sustainable, inclusive, and adaptable. Health coaching has been shown to be an effective approach for improving behavioral changes in self-management. Health coaching combined with telehealth technology has the potential to improve the overall quality of and access to health services.

**Objective:**

This protocol outlines the study design for implementing the Artificial Intelligence for Diabetes Management (AI4DM) intervention. The protocol will assess the feasibility, acceptability, and preliminary efficacy of the AI4DM telehealth platform for people with disabilities.

**Methods:**

The AI4DM study is a 2-arm randomized controlled trial for evaluating the delivery of a 12-month intervention, which will involve telecoaching, diabetes educational content, and technology access, to 90 individuals with diabetes and physical disabilities. The hypothesis is that this pilot project is feasible and acceptable for adults with permanently impaired mobility and type 2 diabetes. We also hypothesize that adults in the AI4DM intervention groups will have significantly better glycemic control (glycated hemoglobin) and psychosocial and psychological measures than the attention control group at the 3-, 6-, and 12-month follow-ups.

**Results:**

The AI4DM study was approved by the university’s institutional review board, and recruitment and enrollment will begin in October 2021.

**Conclusions:**

The AI4DM study will improve our understanding of the feasibility and efficacy of a web-based diabetes self-management program for people with disabilities. The AI4DM intervention has the potential to become a scalable and novel method for successfully managing type 2 diabetes in people with disabilities.

**Trial Registration:**

ClinicalTrials.gov NCT04927377; https://clinicaltrials.gov/ct2/show/NCT04927377

**International Registered Report Identifier (IRRID):**

PRR1-10.2196/31689

## Introduction

### Background

Diabetes mellitus is one of the most common metabolic disorders; it affects approximately 12.2% of the overall adult population in the United States and 25.25% of the population aged above 65 years [1]. One-third of the US adult population is affected by prediabetes. Diabetes can increase the overall risk of premature death and has been linked to several complications, including cardiovascular, renal, and neurological issues. Although diabetes is a major problem among all populations, people with disabilities are more prone to diabetes. According to the Behavioral Risk Factor Surveillance System 2017 report, nearly 1 in 4 people with disabilities are diagnosed with diabetes, whereas approximately 1 in 10 people without a disability are diagnosed with some form of diabetes [2].

Although physical disabilities increase the risk of developing diabetes, an inverse may also occur. Diabetes is associated with a significant increase in the risk of mobility disability [3]. It has been suggested that individuals with diabetes have an increased risk of disability because of multiple factors, such as obesity, depression, and stroke, when compared with individuals with no diabetes [4]. Similarly, numerous studies have indicated that diabetes may lead to disabling disorders, including cardiovascular disease [5], renal dysfunction [6], retinopathy [7,8], and peripheral vascular disease [8]. Older adults with diabetes are also less likely to engage in physical activity (PA) [9], and some are unable to perform minor physical tasks.

In diabetes management, PA, medication adherence, and glucose tracking have been shown to be effective in reducing glycated hemoglobin (HbA_1c_) in both the general population and people with intellectual and developmental disabilities [10-15]. Recent meta-analyses have indicated that diabetes prevention and management programs emphasizing community engagement and tracking of food consumption, especially in the context of a low-carbohydrate diet, were more successful than medications in effective diabetes management [16,17]. Studies that investigated the effect of health promotion initiatives and lifestyle interventions on participants with disabilities also indicate that such efforts can promote healthy diets, regular exercise, and reduce sedentary lifestyle, thereby improving the management of chronic health conditions including obesity and diabetes among individuals with disabilities [15,18,19].

Diabetes education is essential for the treatment and management of diseases. Better education and knowledge can reduce the risk of developing diabetes complications while reducing morbidity and mortality. However, based on the 2014 Behavioral Risk Factor Surveillance System data, 47.3% of adults with disabilities did not receive diabetes education, and 10.86% did not visit health professionals for diabetes in the past year. This problem has been amplified by racial minorities. Low health literacy (LHL) is another major problem among people with disabilities [20,21]. Similarly, older adults, people with less than a high school degree, racial and ethnic minorities, people with low-income levels, and nonnative speakers of English are most likely to experience LHL [22]. Approximately 80 million adults in the United States are estimated to have LHL [23]. People with LHL are more prone to low diabetes knowledge levels [24-27] and limited glycemic control [26], resulting from unhealthy dietary habits, low PA levels, poor medication adherence, and poor blood glucose monitoring [23-27]. People with low literacy skills are not illiterate; however, using plain language is necessary to improve their health literacy and make written and oral information easier to understand [22]. Improving health literacy among participants with LHL would be beneficial for the education, treatment, and management of chronic diseases.

However, people with disabilities face inordinate barriers to achieving a healthy diet and recommended PA levels [28-31]. People with disabilities remain to be one of the least active populations in society [31-33]. Nationwide, 46% of people with disabilities have been categorized as physically inactive [32]. Maintaining a healthy diet is also a challenge for people with disabilities. There are several barriers to this process, such as feeling too tired to cook, organic or healthy, and nutrition foods being expensive, and lack of desire or willpower to cook [29]. All of these issues can be resolved using a systematic approach. However, accessible, inclusive, and adapted diabetes management programs for people with disabilities do not exist. Programs that are not designed with people with disabilities in mind (ie, noninclusive) pose various physical, programmatic, and attitudinal barriers. A few studies have examined diabetes management for people with disabilities, but they have all been singular studies that have not led to the creation of sustainable and scalable diabetes management programs for people with disabilities. For example, the National Center on Health, Physical Activity and Disability created the first *Prevent T2 for ALL*, an inclusive adaptation of the CDC’s *Prevent T2 program*. However, the Prevent T2 program is a diabetes prevention program (not a diabetes management program), and the primary focus is on BMI control, not glycemic control.

As our next step toward diabetes management for people with disabilities, we propose the development of an inclusive telecoaching self-management program for diabetes management in people with disabilities. To make the telecoaching approach sustainable and scalable, artificial intelligence techniques are employed to reduce the time health coaches spend on each participant.

### Objectives

The primary objective of this project is to evaluate the preliminary efficacy and feasibility of an accessible and inclusive artificial intelligence–assisted, individualized, family-focused lifestyle modification intervention (the Artificial Intelligence for Diabetes Management [AI4DM] intervention) for glycemic control in people with disabilities.

We hypothesize that the clinical outcome (HbA_1c_) associated with type 2 diabetes mellitus (T2DM) self-management and self-efficacy outcomes would have a greater effect in the AI4DM intervention arm than in the attention control arm at the 3-, 6-, and 12-month follow-ups. We also hypothesize that the AI4DM intervention is feasible and acceptable for adults with permanent impaired mobility and T2DM, their caregivers, and health coaches and that the fidelity of the program will be maintained. A fidelity monitoring protocol was developed by the research team for the AI4DM study based on the five domains recommended by the National Institutes of Health Behavior Change Consortium [34].

## Methods

### Overview

The AI4DM study is a 2-arm randomized controlled trial. Eligible participants will be randomly assigned to one of two groups: (1) the AI4DM intervention group with telecoaching support and (2) the attention control group. The active intervention will include 6 months of telecoaching followed by 6 months of follow-up and access to the technology but with no telecoaching calls (Table 1). For the study, 4 weeks is considered a month, thereby making the study duration 48 weeks. Study activities involving participants with disabilities will be primarily conducted on the web nationally in the United States.

**Table 1:** Research design.

Group	Enrollment (weeks 1-2)	Pretest (weeks 2-3)	Weeks 4-15^a^	AI4DM^b^ intervention			Posttest (weeks 52-53)
				Weeks 16-27^a^	Weeks 28-51		
AI4DM intervention	✓^c^	✓	Weekly calls and technology access	Biweekly calls and technology access	Only technology access	✓	
Attention control	✓	✓	Weekly courtesy calls	Biweekly courtesy calls	No technology access	✓	

^a^Follow-up data collected at the end of weeks 15 and 27 as well (for both arms of the study).

^b^AI4DM: Artificial Intelligence for Diabetes Management.

^c^Study activity present.

### Explanation for Choice of Comparators

The AI4DM intervention is designed to evaluate the feasibility and preliminary efficacy of a web-based diabetes self-management program for people with disabilities. Participants will either be assigned to the intervention group, which includes telehealth coaching calls, a technology package, and access to diabetes educational content, or an attention control group, which will receive the same number of telecoaching calls at the same frequency as the intervention group. These calls generally serve as courtesy calls or general wellness calls.

### Eligibility Criteria

Eligible participants must meet the following inclusion criteria: (1) a diagnosis of T2DM; (2) an HbA_1c_ level of ≥8%; (3) an age of 18-65 years; (4) individuals living with a permanent physical disability such as spinal cord injury, spina bifida, multiple sclerosis, or stroke and (5) the ability to speak and read English. To screen for permanently impaired mobility, the NHANES Physical Functioning Survey [35] will be used.

Exclusion criteria include (1) the use of insulin medication for diabetes treatment; (2) current enrollment in any diabetes-related intervention; (3) severe cognitive impairment; (4) severe untreated depression in the past 6 months; (5) a major cardiac event in the past 12 months; (6) uncontrolled blood pressure; (7) resting tachycardia; (8) renal failure; (9) severe peripheral neuropathy; and (10) the unavailability of a smartphone.

### Interventions

The total study duration for both groups is 12 months. The protocol includes both groups receiving weekly health coaching calls weekly for 12 weeks, and one biweekly coaching call (every other week) for 12 weeks, for a total of 18 coaching calls. The intervention group, AI4DM, will receive access to home and web-based technology and diabetes-specific educational content through a telehealth app. The attention control group will serve as an untreated comparison group for the AI4DM intervention group. Both groups will also receive 4 HbA_1c_ kits for glucose data collection.

### Study Survey Packets

After enrolling in the study, participants will automatically be emailed a Health Insurance Portability and Accountability Act (HIPAA)–compliant Research Electronic Data Capture (REDCap) link to a survey packet. REDCap is a secure program developed by a collaboration between Vanderbilt University and the National Institutes of Health National Center for Research Resources that manages and stores clinical trial data. This survey packet included a demographic survey and the secondary outcome measures listed in Table 2. Participants will be asked to complete this survey packet 4 times during the 12-month study period (excluding the demographics in the first survey packet). The timepoints included baseline, 3 months, 6 months, and postintervention (12 months).

**Table 2:** Measures of efficacy.

Variables	Instruments	Time points
Glycemic management	HbA_1c_^a^ (clinical and primary outcome)	B,^b^ 3,^c^ 6,^d^ P^e^
Psychological distress	Diabetes Distress Scale	B, 3, 6, P
Quality of life	Diabetes Quality of Life Measure	B, 3, 6, P
Self-efficacy	Diabetes Empowerment Scale	B, 3, 6, P
Family support	Diabetes Family Behavior Scale	B, 3, 6, P
Physical activity	Godin leisure-time exercise questionnaire	B, 3, 6, P
Dietary intake	The UK Diabetes and Diet Questionnaire	B, 3, 6, P
Medication adherence	Medication Adherence Rating Scale	B, 3, 6, P
Telehealth dashboard usability	System Usability Scale & Health Information Technology Usability Evaluation Scale	3, 6, P
Health information technology	The eHealth Literacy Scale	B, 3, 6, P
Medication and dosage	Instruments for controlling medication and dosage during the analysis of the clinical outcome	B, 3, 6, P

^a^HbA_1c_: glycated hemoglobin.

^b^B: baseline.

^c^3: 3-month follow-up.

^d^6: 6-month follow-up.

^e^P: 12-month post–follow-up.

### Welcome Call

After the first survey packet is emailed to the participants, a study team member will call the participants. The purpose of this call is to welcome them into the study and explain the study elements. During the call, the staff will inform the participant that they will receive a package within the next few days (see the section *HbA_1c_ Kits*).

### HbA_1c_ Kits

All participants, regardless of study arm allocation, will receive 4 HbA_1c_ kits. The HbA_1c_ kits will be shipped from a clinical diagnostic laboratory testing company. Research personnel will have access to an account through the testing company website and will send an HbA_1c_ kit 4 times during the study duration: baseline (after the welcome call), 3 months, 6 months, and postintervention (12 months). The HbA_1c_ kit will include a testing kit and return packaging materials. Participants will be asked to complete and return the kit at their earliest convenience.

### Randomization

A randomization sequence will be generated and stored within REDCap, which automatically assigns a participant to the AI4DM intervention group or the attention control group. Randomization will only occur after the baseline survey packet and baseline HbA_1c_ kit are completed.

### Orientation Call

After randomization, a study health coach will be notified from REDCap that a participant has been randomized to one of the study arms and will conduct an orientation call with the participant. The purpose of this call is to introduce themselves to the participants as their primary health coach and to schedule the first weekly coaching call. The first weekly call, regardless of the study arm assignment, will be scheduled for the next calendar week.

### Intervention Package

If a participant is randomized into the AI4DM intervention group, the research staff will coordinate the shipment of a technology package that will be delivered to the participant’s residence within 1 to 2 days after randomization. The contents of this intervention package will include (1) an Amazon Echo, (2) a Fitbit Flex device, and (3) a wireless glucometer. Participants will also be given supplemental instructions for signing in to the mobile health (mHealth) app and device setup instructions. This package is used for intervention delivery and not for outcome measurements.

### AI4DM Intervention Group Coaching Calls (First 12 Weeks)

Coaching calls for the intervention group will be guided by a prepared outline of diabetes-related content that will be delivered to the participant through the mHealth app. This content will be delivered at the beginning of each week before the coaching call. The educational content is derived from using the Partnership to Improve Diabetes Education program and delivered to the participant as multimedia content [36]. Other health-related areas covered during the coaching call will include nutrition and eating habits, exercise and PA, glucose monitoring, and medication adherence. Questions outside the scope of the health coaching content will be provided to the study clinician or registered dietician. If the study team determines that the questions require collecting a participant’s detailed medical profile, the participant will be guided to communicate with their primary care physician. Finally, the health coach will address other health-related questions that the participant may have. For each call, the health coach will be able to record notes in the telehealth app regarding topics discussed with the participant for future calls, as needed. Coaching calls are expected to last for up to 60 minutes.

### AI4DM Intervention Group Coaching Calls (Second 12 Weeks)

Biweekly coaching calls during the following 12 weeks will cover nutrition and eating habits, exercise and PA, glucose monitoring, medication adherence, general well-being, and Partnership to Improve Diabetes Education content, as needed.

### Attention Control Group Coaching Call

The coaching content for the attention control group will refrain from any diabetes-specific content; rather, the health coach will only focus on general well-being. This will apply to all phone calls during weekly and biweekly coaching calls.

### Exit Interviews

After the study duration, up to 30 participants from the AI4DM intervention group will be contacted for a follow-up interview to obtain their experiences and feedback with the AI4DM telehealth platform. These interviews will be conducted virtually.

### Outcome Measures

Primary and secondary outcome measures will be assessed at baseline, 3 months, 6 months, and postintervention (12 months). The primary clinical outcome measure is HbA_1c_ with the secondary outcome measures described in Table 2.

### Data Collection Methods

#### HbA_1c_ Collection

HbA_1c_ kits will be distributed and received by a clinical at-home laboratory testing service. Study personnel will use the company’s web-based resources to ship at-home test kits to all participants. The test kit will contain all necessary items to collect, prepare, and mail dried blood specimens to the laboratory for testing. Upon receiving the kit, the testing company will report the values through their network, and study personnel will obtain the values and record them directly into REDCap.

All other measures, including demographic data and secondary outcome measures, will be retrieved and automatically stored through electronic surveys delivered by REDCap. All self-reported items within the questionnaires will be required to prevent missing data. The questionnaire packet will be delivered electronically to the participants at 4 time points during the study (Table 2).

#### Psychological Distress

To measure psychological distress, participants will complete the Diabetes Distress Scale (DDS). This 17-item questionnaire assesses diabetes-related emotional distress using Likert-style questions for the previous month. The DDS has been shown to be a valid and reliable tool for measuring diabetes-related emotional distress [37,38].

#### Quality of Life

Participants will complete the Diabetes Quality of Life Measure, which is a 15-item questionnaire asking about perceptions of one’s ability to manage their diabetes in conjunction with other life areas [39].

#### Self-efficacy

To measure psychosocial self-efficacy in diabetes management, participants will complete the Diabetes Empowerment Scale Short Form (DES-SF). The DES-SF is an 8-item questionnaire asking about attitudes toward diabetes and the ability to successfully manage diabetes. The DES-SF has been shown to be a valid and reliable measure of psychosocial self-efficacy [40].

#### Family Support

Participants will complete the Diabetes Social Support Questionnaire, which is a 52-item questionnaire that asks questions related to familial support for individuals with diabetes [41]. This questionnaire was originally designed for adolescents with diabetes; however, the original version was modified to reflect questions appropriate for adults. Thus, the Diabetes Social Support Questionnaire used will be a 33-item questionnaire.

#### Physical Activity

To measure PA, participants will complete the Godin Leisure-Time Exercise Questionnaire. This short questionnaire asks three questions related to strenuous, moderate, and light exercises performed during the past seven days [42].

#### Dietary Intake

Participants will complete the UK Diabetes and Diabetes Questionnaire to assess nutrition and dietary behaviors within the past month [43]. This 24-item questionnaire includes foods that are common in the United Kingdom; therefore, those items that are specific to the United Kingdom will be replaced with items equivalent in the United States to reduce confusion for participants.

#### Medication Adherence

To measure medication adherence, participants completed the Medication Adherence Rating Scale. The Medication Adherence Rating Scale is a general, 10-item binary questionnaire that asks questions related to regularly taking medication [44].

#### Health Information Technology Literacy

Participants will complete the eHealth Literacy Scale, which is an 8-item survey that measures perceived knowledge, comfort, and skill when using technology to address overall health [45].

#### Telehealth Dashboard Usability

To measure the usability of the telehealth dashboard, participants will complete the System Usability Scale and the Health Information Usability Evaluation Scale. These Likert scales include items asking about the dashboard’s effectiveness (ability to complete tasks), efficiency (level of dashboard use), and satisfaction (subjective reactions to the dashboard) [46,47].

#### Medication and Dosage

Participants will be asked to provide current medications and dosages throughout the study duration to control medication data during the statistical analysis.

### Feasibility Measures

This study will obtain measures related to the feasibility and acceptability of the AI4DM intervention on diabetes self-management. Measures will include process, resource, scientific, and management feasibility outcomes. All elements of intervention delivery will be collected, including but not limited to adherence, retention, attrition, coach and participant communication needs, staff preparation, and adverse events. Obtaining these measures will inform future considerations in the delivery of a web-based diabetes self-management program for people with disabilities.

### Participant Timeline

The study duration for those enrolled in the study will be 12 months, regardless of study arm allocation.

### Sample Size

A total of 90 participants will be enrolled. The sample size for this protocol is based on a primary analytic strategy of analysis of covariance, a two-sided test, type 1 error rate of 0.05, and intention-to-treat analysis with multiple imputations. Assuming a correlation between baseline and follow-up outcomes of at least 0.7, we will have 80% power to detect an effect size of 0.65.

### Recruitment

The entire recruitment will be conducted on the web nationally through the National Center on Health, Physical Activity and Disability website and its associated social media. Through these mechanisms, interested individuals will be directed to a landing website page that will have promotional media content and information about the study. From there, participants will be directed to a separate link through HIPAA-compliant REDCap and complete a screening eligibility form. If deemed eligible, the participants will be sent an electronic consent form to be completed using the HIPAA-compliant REDCap. On the basis of our recruitment methods, we expect that approximately 180 individuals will be screened for eligibility.

### Allocation

Participants will be randomized to the two study arms in a 1:1 allocation ratio using a computer-generated randomization procedure. The allocation sequence will be implemented after the baseline surveys, and the baseline HbA_1c_ kit has been completed by the participant. The computer-generated randomization process will be implemented in REDCap after the data from the baseline surveys and HbA_1c_ kit are entered into the REDCap database. This will trigger the allocation procedure to randomly assign participants to one of the two study arms. The health coach will be notified about the randomization through the telecoaching dashboard and will inform the participants about their randomization into the AI4DM intervention or the attention control arm during the orientation call.

### Blinding (Masking)

The principal investigator and primary statistician will be blinded to the randomization of the participants into the study arms. All other study staff will be unblinded for purposes of recruitment, consent, orientation calls, welcome calls, coaching calls, and intervention package shipping. There are no circumstances in which the principal investigator or statistician will become unblinded during this protocol.

### Data Management

All data collected will be entered directly into REDCap. Participants will be assigned a subject number upon enrollment in the study. Only approved study personnel will have access to the REDCap program, where data will be stored. Data checking will involve confirming that surveys, questionnaires, and HbA_1c_ values are completed.

### Statistical Methods

All data will be examined for normality violations, outliers, errors, and patterns of missing values. For primary and secondary outcome measures, the sample and variables will be described using frequency distributions and appropriate summary statistics. Randomization across the study arms will be checked. If any baseline variable differs by group, it will be included as a covariate in subsequent analyses. Our primary analysis for the hypothesis that adults in the AI4DM intervention group will result in better glycemic control than the attention control group will be tested using analysis of covariance with baseline measurement as a covariate. Multiple imputations will be used to impute missing data. The study data will also explore the pattern and missing mechanism, and additional sensitivity analyses will be performed to test parametric assumptions as well as assumptions of missingness. Outliers and normality of quantitative variables will be evaluated. This study is powered to enable the estimation of effect sizes. Analysis of the clinical outcome (HbA_1c_) will control for the impact of medication and dosage during the intervention period.

### Data Monitoring

This project is a pilot feasibility and efficacy study that has duration of 12 months and only has minimal risks. Therefore, we believe that a data monitoring committee is not required. The principal investigator will be responsible for protocol fidelity and data collection throughout the study. The biostatistician will oversee data analysis in preparation for publication.

### Harms

The AI4DM study will monitor all adverse events. Adverse events will be assessed for severity and causality and will be reported to the institutional review board and all other relevant regulatory bodies as needed.

### Auditing

All elements of the study protocol will be evaluated at regular intervals (ie, weekly, monthly, or quarterly) to ensure proper and consistent adherence to the study design and implementation. Elements of auditing will include checklists, coaching call logs, audio recordings or coaching calls, content resource banks, telehealth platform reviews and event logs, review of participant food, PA, medication, glucose entries, time spent on the platform, and team meetings to discuss participant progress and protocol adherence.

### Research Ethics Approval

All proposed elements of this protocol will be approved by the university’s institutional review board before beginning the study.

### Protocol Amendments

Any changes deemed necessary by the principal investigator will be submitted as amendments to the university’s institutional review board and will only make changes after approval.

### Consent or Assent

All consent processes will be conducted on the web through predetermined screening questionnaires provided through the landing website and REDCap link. If a participant is deemed eligible through the web-based process, an electronic consent form will be sent to the participant to complete. Only approved study staff will have access to complete records of the consent form.

### Confidentiality

Personal information will be collected in this study. All personal information shared, such as demographic details, will be collected and stored through HIPAA-compliant REDCap. Only approved study staff will have access to securely stored personal information. All information will be subject to the university’s institutional review board and the policies of affiliated entities surrounding confidentiality.

### Declaration of Interests

There are no declarations of interests for all study staff for this protocol.

### Ancillary and Posttrial Care

This is not applicable. No provisions for posttrial care are included in the study.

### Dissemination Policy

The results of this study will be disseminated publicly through publications in peer-reviewed journals and presented at regional and national conferences.

### Appendices: Biological Specimens

Blood samples will be collected for HbA_1c_ analysis at 4 different time points (baseline, 3-month, 6-month, and 12-month). The laboratory testing service will be responsible for mailing, receiving, and processing completed HbA_1c_ kits. The study personnel will not interact or have access to blood samples at the testing laboratory.

## Results

The AI4DM protocol has been approved by the university’s institutional review board and has been registered at ClinicalTrials.gov (trial number: NCT04927377). The mHealth app (dashboard and educational content) and landing website are currently under development by the study technical support team, and research personnel are developing the database (REDCap) that will store data and communication mechanisms (ie, emails and text notifications). We estimate that enrollment for the study will begin in October 2021.

## Discussion

Although diabetes mellitus is one of the most common metabolic disorders affecting millions in the US population, this disorder has become more prevalent in people with disabilities [2]. Compared with nondisabled individuals, people with disabilities experience higher rates of diabetes, which leads to several health-related complications. In addition, people with disabilities experience and face barriers that prevent the ability to successfully manage their diabetes through exercise and healthy eating [28-31]. Therefore, the development of an inclusive diabetes management program for people with disabilities is warranted.

As multiple barriers exist that would hinder a person’s ability to successfully manage diabetes, AI4DM seeks to create a sustainable, scalable, accessible, and inclusive diabetes management program for people with disabilities, which currently does not exist. This study will examine the feasibility and preliminary efficacy of AI4DM for glycemic control (HbA_1c_) for people with disabilities using mobile and web-based apps, telehealth coaching sessions, and diabetes-related educational multimedia content. The AI4DM study will also focus on quality-of-life measures during the intervention and will include qualitative interviews following the intervention to provide a holistic evaluation of the intervention program as a viable mode of diabetes management. Next, to reduce barriers experienced by people with disabilities, the AI4DM program will be conducted completely on the web with all coaching sessions taking place over the telephone, thereby allowing all participants to manage their diabetes in a home environment. Finally, the results of this feasibility study will inform how web-based chronic disease management programs can be improved for future studies.

## Appendix

Multimedia Appendix 1 Peer-reviewer report from the National Institute on Disability, Independent Living, and Rehabilitation Research.

## Abbreviations

AI4DM: Artificial Intelligence for Diabetes Management
DES-SF: Diabetes Empowerment Scale Short Form
HbA_1c_: glycated hemoglobin
HIPAA: Health Insurance Portability and Accountability Act
LHL: low health literacy
mHealth: mobile health
PA: physical activity
REDCap: Research Electronic Data Capture
T2DM: type 2 diabetes mellitus
