# Supplementary material for: Disability-Inclusive Diabetes Self-management Telehealth Program: Protocol for a Pilot and Feasibility Study
Source: JMIR Res Protoc. 2021 Sep 10;10(9):e31689. doi: 10.2196/31689 (PMC8463947; doi:10.2196/31689)
Supplement: Supplementary file 1 [file resprot_v10i9e31689_app1.pdf]

September 9, 2019

Mohanraj Thirumalai, PhD
1720 2nd Avenue South
AB 1170
SHPB 590E
Birmingham, AL 35294-0111

Application Name: ***University of Alabama at Birmingham***
Application Number: ***IFDV19000121***

Dear Dr. Thirumalai:

Congratulations! Your application to the National Institute on Disability, Independent Living, and Rehabilitation Research (NIDILRR) for consideration under the Fiscal Year 2019 ***Field Initiated Projects Grant Competition (Research)*** has been selected for funding.

Each application was objectively reviewed, discussed, and scored by a peer review panel. Panel members evaluated applications according to the criteria published in the funding opportunity announcement. I am enclosing a copy of the comment sheets prepared by the peer reviewers. If you wish to discuss the peer review process, the reviewers' comments or any related matters pertaining to your application or NIDILRR's FIP Program, please contact Theresa San Agustin, MD at Theresa.SanAgustin@acl.hhs.gov.

Thank you for your interest in the Field Initiated Projects Grants. I look forward to your continued interest in NIDILRR grant programs.

Sincerely,

Kristi Wilson Hill

Kristi W. Hill, PhD
Acting Director
National Institute on Disability, Independent Living, and Rehabilitation Research

Enclosures

Applicant Final Panel Summary Report

Average Score: 94.40

Application Number: IFDV19000121
Application Name: UNIVERSITY OF ALABAMA AT BIRMINGHAM
State: AL City: Birmingham

Criteria Name(Max Score)

1. Importance of the problem (20 Points)
2. Design of development activities (50 Points)
3. Plan of evaluation (5 Points)
4. Project staff (15 Points)
5. Adequacy and accessibility of resources (10 Points)

TOTAL: 100

Summary of Panel Discussion

Strength:

- Applicant provided a strong sense of scientific context as evidenced by credible statistics supporting the documented problem, detailed methodology, and appropriate use of sample size and trials.
- Applicant thoroughly depicted the need and provided a good rationale for proposed project.
- Applicant clearly depicted an underlying theoretical framework throughout the proposal.
- Applicant clearly described how the infrastructure and content is planned to be inclusive and accessible, therefore depicting how the project is beneficial to the target population.
- Applicant provides a full complement of the necessary experienced personnel with extensive experience.
- Applicant presented a strong case of periodic assessments (e.g., weekly, quarterly) and encompasses feedback loop for improved performance.
- Applicant demonstrated how the proposed project is innovative and presents a successful likelihood that the results will have a transformative effect on the target population.
- Applicant clearly depicts the engagement of stakeholders as evidenced by the use of a participatory design approach.
- Applicant provides great recruitment strategies and remedies to address possible pitfalls.
- Applicant clearly depicts standard policy of non-discrimination and encourages/actively seek applications from underrepresented groups.
- Applicant clearly depicts adequate facilities and resources that are accessible and ADA compliant

Weakness:

- Applicant was limited in fully addressing personal and social support barriers that the target population may encounter.
- Applicant was limited in accounting for access and usage issues (e.g., technology & cost) which could hinder broad application and generalizability.
- Applicant was limited in providing a comprehensive description of the logic model application.
- Applicant was unclear in describing the role of the caregivers as the proposal stems around the promotion of self-managed health activities.
- While the developmental activities are well conceived and organized, the feasibility of accomplishing all of the tasks in three years appears unrealistic in scope.

General Feedback:

N/A

Technical Review Form : 1

Criteria Name(Max Score) : Actual Score

1. Importance of the problem (20 Points) : 20
2. Design of development activities (50 Points) : 45
3. Plan of evaluation (5 Points) : 3
4. Project staff (15 Points) : 15
5. Adequacy and accessibility of resources (10 Points) : 10

TOTAL: 93 / 100

Rationale

Scoring Criteria

Criterion 1: Importance of the problem

Strength:

Page: 1

The applicant defines need for diabetes type 2 control among people with physical disabilities and how they may be underserved in the absence of self-management competencies with assistive technologies. The case for need is clearly made from a review of the literature. The importance of the problem is also well articulated in view of the disproportionate prevalence of diabetes among adults with physical disabilities.
The proposal is consistent with the primary purpose of the Rehabilitation Act to enable independent living with disability and for community participation with good health and function. The applicant does not address how the project aligns with NIDILRR priorities or long-term plans.

Benefits to the participants include availing training support using smart technologies for managing their type 2 diabetes.

Weakness:

None

General:

None

Criterion 2: Design of development activities

Strength:

Page: 1

The proposal demonstrates awareness of the state-of-the-art in several ways: compensate review of current programs for diabetes type 2 management involving coaches, person to person, on lien and virtual. The applicant make a strong case for the relative advantage of artificial intelligence systems compared to currently available: accessibility and person-oriented.
The proposed project also demonstrates knowledge of appropriate concepts in artificial intelligence with telecoaching, including proven systems for their use. The proposal seeks to apply sociocognitive theory to understanding the personal and social choices people make in their health proportion.

Appropriate samples and procedures are proposed for all project activities including focus group discussion, accessibility and usability testing. Moreover, the proposal is based on studies completed by the applicant that proofed some of the tools for its implementation presently available and accessible online.

The project is proposed to implement in with a community provider of physical and health function services with a long history in the field and a strong partnership with the applicant organization. Input from people with disabilities and stakeholders is to be gathered at all stages of the project implementation.

The proposal identifies the project as a proof-of-concept and proof of product. This is an appropriate designation for the activities to be implemented, particularly since the main tools for the product development are in place already.

Weakness:

Page: 4

The planned exclusion from study of all people currently involved with a diabetes is probably unrealistic as most people would be on some type of program, and perhaps not telehealth. It is not clear what the role of caregivers will be in a self-managed health control, how involved caregivers will be and what how the intervention will be different for those without caregivers. The mix of physical disabilities to be include is too broad to ensure equity in user demand and response to allow for reliable findings.

General:

None

Criterion 3: Plan of evaluation

Strength:

Page: 5

The proposal presents a plan of work using Asana for activity scheduling.The project will host evaluation meetings to inform and track ongoing activities.

Weakness:

Page: 6

Use of Asana is not the same as a plan of evaluation with inbuilt and explicitly described tools for evidence to improve the performance of the project through the feedback from implementation activities.

General:

None

Criterion 4: Project staff

Strength:

Page: 7

Project staff are eminently qualified in all aspects of the project with several of the having either designed a tool to be adapted for this study and/or implemented previous mHealth and or health coaching projects aimed at diabetes control. The key personnel are extensively published and with extramural funding to study diabetes control with vulnerable populations. The work there are proposing to do is a logical next step.

Weakness:

None

General:

None

Criterion 5: Adequacy and accessibility of resources

Strength:

Page: 8

The applicant organization has the resources to successfully implement the proposed project. They also have diversity among staff and accessible physical and data/information infrastructure, including accessible buildings, workspaces, and presentation of information in alternative formats (electronic and print media).

Weakness:

None

General:

None

Technical Review Form : 2

Criteria Name(Max Score) : Actual Score

1. Importance of the problem (20 Points) : 20
2. Design of development activities (50 Points) : 47
3. Plan of evaluation (5 Points) : 5
4. Project staff (15 Points) : 15
5. Adequacy and accessibility of resources (10 Points) : 10

TOTAL: 97 / 100

Rationale

Scoring Criteria

Criterion 1: Importance of the problem

Strength:

Page: Page 77 of 184

The application clearly highlights the Director's priority as defined by the extensive discussion, specifically addressing the impact of physical inactivity in its target population, inclusive of individuals living with disabilities and type 2 diabetes.

Page: Page 77-78 of 184

The applicant clearly describes the need and target population. For example, the applicant provides recently published studies to support the need: " Diabetes mellitus (DM) is one of the most common metabolic disorders affecting about 12.2% of the overall US adult population and 25.25% of the population over the age of 65." Additionally, per a 2017 BRFSS report, "23.6% of people with disabilities are diagnosed with diabetes, in contrast 9% of people without a disability are diagnosed with diabetes."

Page: Page 86 of 184

The applicant clearly describes how it meets the Rehabilitation Act and success of legislation, describing crucial and critical points that lend to long-term self-management and health promotion, indicating that without self-management programs, secondary concerns such as the long-term implications to society; specifically, the individual impact to individuals living with disabilities and their ability to access and sustain gainful employment and self-care.

Page: Page 86 of 184

The applicant clearly describes and illustrates the beneficial impact (i.e. technologically advanced scalable infrastructure) in the target population, and includes a broader discussion of its long-term design. The proposed intervention would be accessible to all anyone via its tele-coaching modalities.

Page: Page 87-88 of 184
The application clearly indicates the successful expectation of the program to include improvements in overall health status, specifically stating that the primary objective of this intervention is to, "develop and pilot test an accessible and inclusive Artificial Intelligence (AI)-assisted, individualized, family-focused lifestyle modification intervention (AI4DM) for glycemic control in people with disabilities." The proposal references a clear and time-framed "logic model."

Weakness:

None

General:

Page: Page 87-88 of 184
The application references a logic model for clarity of strategy, outcomes and time-frames. The logic model does not clearly incorporate several components to fully access the proposed outcomes and alignment (e.g., inputs, outputs).

Criterion 2: Design of development activities

Strength:

Page: Page 103-10 of 184
In this one-of-a-kind intervention, the applicant fully describes the current and final stages of reseach (clinical trials). The current sample will be recruited via an external recruitment source - extended to 4000 individuals livng with disabilities and chronic conditions. Eligibility criteria is clearly defined to include seven points of entry including: " 1) Diagnosis of T2DM; 2) HbA1c ≥ 8% (for Aim 2 only); 3) 18 to 65 years of age; 4) living with a permanent physical disability such as SCI, spina bifida, multiple sclerosis, stroke (NHANES Physical Functioning Survey123 will be used to screen for permanent impaired mobility for Aim 2) ; 5) have the ability to converse in and read English." The exclusion criteria is clearly defined. Proposed activities clearly align with the goal of the proposed project. For example, the applicant proposes the use of Cognitive-Behavioral Therapy, specifically leveraging the expertise of staff to engage Participants and caregivers and utilize CBT techniques during counseling.

Page: Page 115 of 184
The applicant clearly states that activities will be conducted physical location (Lakeshore Foundation) where activities such as interviews and usability testing for example, will be conducted among users with disabilities.

Page: Page 88-9 of 184
The applicant clearly infers higher cost comparisons of prior interventions based on a systematic review of 118 interventions, stating greater barriers such as social and environmental factors that impede(d) full participation. Further, the applicant succinctly details costs of nearly of nearly $1,400/annually to participate for other interventions.

Page: Page 92 of 184
The applicant fully describes its innovative program which integrates artifical intelligence for a more comprenhisive approach to care. For example the applicant states, that in order to address various deficiencies previously described discussed, it proposes to use a blended approach, "that combines human telecoaching and

mHealth technologies while including caregiver-specific features to promote T2DM self-management for people with disabilities."

Weakness:

Page: (Information not found in the application review)

The application does not clearly descibe "how" it proposes to engage key stakeholders in the implementation of the proposed project or in its development and design, i.e. feasibility of flexible engagement for data consistency and transparency.

Page: (Information not found in the application review)

The application does not include a clear discussion of cost comparisons for full consideration of the proposed technology.

General:

Page: Page 116 of 184

This development proposal is planned to overlap between the Proof of Concept (POC) andProof of Product (POD) stages of NIDILRR's Stages of Development.

Page: page 90 of 184

The applicant describes similar technologies currently on the market.

Criterion 3: Plan of evaluation

Strength:

Page: Page 118-19 of 184

The Director clearly considers the quality of the plan of evaluation as determined by the application's included discussion of periodic feedback from stakeholders to include facilitators and consumers. For example, the applicant proposes to incorporate stakeholders and their feedbback into response loop to improve processes. Weekly and quarterly meetings clearly threaded in into scheduling in addition to Consumer Advisory meetings aimed at building content and soliciting feedback.

Weakness:

None

General:

None

Criterion 4: Project staff

Strength:

Page: 0

The applicant clearly considers the quality of project staff by considering applications for employment from persons who are members of groups that have traditionally been underrepresented based on race, color, national origin, gender, age, or disability. For example, the narrative suggests that the project personnel clearly represent individual from diverse backgrounds, following federal and state guidelines regarding affirmative action. The application fully identifies two individuals who also serve as representives from the targeted community who also serve as key personnel.

Page: Page 120-1 of 184

The applicant clearly considers the extent to which key personnel and other key staff have appropriate training and experience in disciplines required to conduct all proposed activities. For example, the PI is also the Co-Director of the RERC on Technologies to Promote Exercise and Health among People with Disabilities, a center that focuses on various technology based physical activity interventions for people with disabilities.

Weakness:

None

General:

Page: (Information not found in the application review)

The application does not clearly define a process for hiring individuals from underrepresented communities.

Page: Page 126 of 184

One full-time staff member is responsible for grants administration and general office administrative tasks for all research projects in the PI's department (Health Services Administration). This staff member will help the PI with all pre and post award activities.

Criterion 5: Adequacy and accessibility of resources

Strength:

Page: Page 120 of 184

The Director considers the quality of project staff, considering applications for employment from persons who are members of groups that have traditionally been underrepresented based on race, color, national origin, gender, age, or disability. For example, the narrative suggests that the project personnel clearly represent individual from diverse backgrounds, following federal and state guidelines regarding affirmative action. The application fully identifies two individuals who also serve as representives from the targeted community who also serve as key personnel.

Page: Page 127 of 184

The applicant highlights clear access to resouces, noting that the organization has several staff with disabilities and where necessary, provides modifications to the workspaces (e.g., specialized keyboards). Project materials will be available, by request, electronically or in large print, Braille, audiotape, CD, videotape, or DVD formats.

Page: Page 31 of 184

Innovative accessibility methods described include customizable weight scales for meeting needs of participants and staff.

Weakness:

None

General:

None

<u>Technical Review Form : 3</u>

Criteria Name(Max Score) : Actual Score

1. Importance of the problem (20 Points) : 20
2. Design of development activities (50 Points) : 45
3. Plan of evaluation (5 Points) : 5
4. Project staff (15 Points) : 15
5. Adequacy and accessibility of resources (10 Points) : 10

TOTAL: 95 / 100

Rationale

Scoring Criteria

Criterion 1: Importance of the problem
Strength:

<u>Page: 1, 5, 9</u>

Applicant clearly provided credible statistics supporting the documented problems associated with the target population.

Applicant thoroughly depicted the need and provided a good rationale for proposed project.

Applicant clearly depicted an underlying theoretical framework throughout the proposal.

Applicant clearly described how the infrastructure and content is planned to be inclusive and accessible, therefore depicting how the project is beneficial to the target population.

Weakness:

None

General:

None

Criterion 2: Design of development activities
Strength:

Page: 12, 22, 27-30, 38-39

Innovative and presents a successful likelihood that the results will have a transformative effect on the target population.

Has a strong sense of scientific context and employs appropriate use of sample size and trials.

Provides great recruitment strategy and remedies to address possible pitfalls.

Uses a participatory design approach and clearly depicts engagement of stakeholders from conception.

Evidence of theoretical framework depicted throughout the proposal.

Strongly identified stage of development.

Weakness:

Page: 29-30

While the developmental activities are well conceived and organized, the feasibility of accomplishing all of the tasks in three years may be unrealistic in scope.

General:

None

Criterion 3: Plan of evaluation

Strength:

Page: 41-42

Strongly depicts plan of operation and how key stakeholders will be involved in the evaluation process.

Innovative in that it uses an online project/task management system (ASANA) that will break down each detail of the evaluation plan. Greatly details how the staff will be required to regularly update the system with progress and any hurdles faced.

Demonstrated effectiveness of periodic assessments (e.g., weekly, quarterly) and encompasses feedback loop for improved performance.

Weakness:

None

General:

None

Criterion 4: Project staff

Strength:

Page: 43, 45

Clearly depicts standard policy of non-discrimination and encourages/actively seek applications from underrepresented groups. The applicant particularly identifies two project personnel who have disabilities.

The applicant provides a full complement of the necessary experienced personnel with extensive experience.

Weakness:

None

General:

None

Criterion 5: Adequacy and accessibility of resources

Strength:

Page: 48-50

Clearly depicts adequate facilities and resources that are accessible and compliant (e.g.,. very accessible technological resources, adheres to web content guidelines).

Weakness:

None

General:

None

Technical Review Form : 4

Criteria Name(Max Score) : Actual Score

1. Importance of the problem (20 Points) : 19
2. Design of development activities (50 Points) : 47
3. Plan of evaluation (5 Points) : 5
4. Project staff (15 Points) : 15
5. Adequacy and accessibility of resources (10 Points) : 9

TOTAL: 95 / 100

Rationale

Scoring Criteria

Criterion 1: Importance of the problem

Strength:

Page: 2

2.i. 47.30% of the adults with disability did not receive diabetes education and 10.86% did not visit health professionals for diabetes in the past year.

Page: 9

2.ii. The approach reflects the Guidelines and Criteria for the Implementation of Community-based Health Promotion Programs for Individuals with Disabilities, including 1) use of an underlying theoretical framework (Social Cognitive Theory),
2) implementation of process evaluation, 3) use of disability-appropriate outcome measures, 4) active involvement of people with disabilities in intervention development and implementation, 5) support of personal beliefs, practices, and values of people with disabilities, 6) consideration of accessibility and barriers to program participation, and 7) sensitivity to financial constraints often experienced by people with disabilities.

Page: 9,10

2.iii. Successful completion of the first aim will create a technologically advanced scalable infrastructure, which will enable people with disability and diabetes to be telecoached and trained for self-management behavior. Completion of the second aim will shed new scientific knowledge on the usage of telecoaching for glycemic control in people with permanent impaired mobility and will also lay the foundation for future studies to focus on other forms of disabilities such as sensory and intellectual disabilities.

Weakness:

Page: 1-10

A range of physical and personal barriers are cited but their need is not incorporated into the benefit of the project.

General:

Page: 1

One-third of the US adult population is affected with prediabetes. DM can increase the overall risk of dying prematurely and has been linked to several complications, including heart attack, stroke, kidney failure, leg amputation, vision loss, and nerve damage. In 2015, DM was listed in 79,535 death certificates as the underlying cause of mortality and 7.2 million hospital discharges in 2014 included diabetes as the diagnosis. This makes DM the seventh leading cause of death only in the United States (incidence rate, 24.7 per 100,000 persons).

Criterion 2: Design of development activities

Strength:

Page: 12-15

2.i.A. Good review of the state of the art for current technology solutions with their limitations.

Page: 27

2.i.B. The purpose of this proposal is not creating new core technologies, but rather adapt successful technologies for the purpose of T2DM management in people with disabilities. The above technologies have all been researched and implemented, while we only intend to adapt and use them for our said purpose.

Page: 28

2.i.C. A total of n = 110 participants and their caregivers (dyads) (n = 20 for Aim 1 and n = 90 for Aim 2) will be recruited. Aim 1 will also recruit potential health coaches (n = 20).

Page: 38

2.i.D All formative development activities such as interviews, usability testing etc., involving users with disabilities and health coaches will be conducted at Lakeshore Foundation.

Page: 39

2.i.E. Aim 1 of this project involves a participatory design approach and will systematically elicit feedback from people with disabilities and T2DM, their caregivers and stakeholders.

Page: 39

2.i.F. This development proposal is planned to overlap between the Proof of Concept (POC) and Proof of Product (POD) stages of NIDILRR's Stages of Development.

Weakness:

Page: 21

2.i.B The monitoring of blood glucose should be more detailed.

Page: 28

2.i.C. A sample of 110 seems excessive for a development project.

General:

Page: 10

The primary objective of this project is to develop, and pilot test an accessible and inclusive Artificial Intelligence (AI)-assisted, individualized, family-focused lifestyle modification intervention (AI4DM) for glycemic control in people with disabilities

Criterion 3: Plan of evaluation

Strength:

Page: 41,42

1.i. Weekly Meetings. We will use weekly research meetings to monitor weekly progress and plan for the upcoming week. Quarterly Meetings. Quarterly meetings will be scheduled with all Co-Is and staff to assess the overall project progress and any course correction needed to the chosen strategies.

Page: 42

1.ii. Specific steps to improve the performance of the project include: (a) ongoing evaluation of progress through regular meetings, (b) adherence to the work plan, and (c) implementation of corrective measures.

Weakness:

None

General:

Page: 39,40

The plan is designed to regularly evaluate progress towards achieving the project goals, using key indicators related to development of the artificial intelligence power telecoaching infrastructure, inclusive diabetes education content, recruitment, enrollment and attrition rates, intervention delivery, and performance of data collection, processing, and analytic activities.

Criterion 4: Project staff

Strength:

Page: 43

2. The applicant organization, the University of Alabama at Birmingham (UAB), has a standing policy of non-discriminatory employment and of admission of students from traditionally underrepresented groups including minorities, women and people with disabilities.

Page: 43-48

3. Key staff have appropriate training and experience.

Weakness:

None

General:

None

Criterion 5: Adequacy and accessibility of resources

Strength:

Page: 50

2.ii. All participant or public facing activities of the project will be conducted at Lakeshore Foundation. Lakeshore Foundation maintains one of the nation's premier universally designed facilities for people with physical disabilities and chronic health conditions.

Weakness:

Page: 48-50

2.i. It is assumed that the applicant will provide adequate facilities, etc. but a commitment was not stated.

General:

Page: 48

The project's activities will draw on the extensive and highly interrelated research, educational and clinical programs at the University of Alabama at Birmingham and Lakeshore Foundation. The UAB campus includes ten schools and is the largest university- based medical center in the State of Alabama. Six schools comprise the UAB Academic Health Center. One of those schools is the School of Health Professions. The School of Health Professions (SHP) at UAB is one of the largest health professions schools in the United States. The School employs 216 paid faculty and staff.

Technical Review Form : 5

Criteria Name(Max Score) : Actual Score

1. Importance of the problem (20 Points) : 19
2. Design of development activities (50 Points) : 44
3. Plan of evaluation (5 Points) : 4
4. Project staff (15 Points) : 15
5. Adequacy and accessibility of resources (10 Points) : 10

TOTAL: 92 / 100

Rationale

Scoring Criteria

Criterion 1: Importance of the problem

Strength:

Page: 86

The director considers the importance of the problem and centers the problem among the target population of individuals with disabilities also living with diabetes.

There is a clear tie from the project to the spirt of empowerment and addressing health and function in the Act.

Weakness:

Page: 94

There is lots of discussion about barriers, little is discussed regarding how the project will evaluate and/or alleviate such barriers experienced by individuals. More clarity is needed for addressing both personal (e.g., lack of social support, physical function) and environmental (e.g., access, physical environment) barriers.

General:

None

Criterion 2: Design of development activities

Strength:

Page: 97; 104; 116

The blending of AI-assisted dietary planning, tele-coaching, sensor based PA tracking and more is novel and exciting.

Recruitment, materials, and administration of the project lends well to the participatory design method.

Flexibility in stakeholder involvement describes individuals to participate in this project without caregivers.

Weakness:

Page: 96; 106; 114; 112

When talking about caregivers, this project seems to assume that these caregivers are cohabitating and have both the ability and the bandwidth to support these individuals in the event that the individual chooses to not involve a health coach.

Further, the analyses don't seem to consider participants who choose to have flexible stakeholder involvement.

Lack of clarity among how existing health monitoring will be integrated into the participants' use with the AI-device. For example, monitoring of blood glucose could be more integrated into the design, many individuals living with diabetes have a system that monitors their glucose already. It seems redundant to have them tell the AI-assisted device.

General:

None

Criterion 3: Plan of evaluation

Strength:

Page: 107; 111; 116

Step by step plan of how to conduct evaluation at each stage of the project. This includes iput.

Weakness:

Page: 107-112

There is lots of discussion about the inclusion of evaluation of the program including telephone interviews with participants, it is less understood about how these evaluations will inform changes to the program.

General:

None

Criterion 4: Project staff

Strength:

Page: 120; 151

The principal investigator, staff and subcontractors seem to be as diverse and inclusive as possible with a wide breath of experience and training.

Weakness:

None

General:

None

Criterion 5: Adequacy and accessibility of resources

Strength:

Page: 121; 82
The facilities are described to be fully accessible.

In the intervention specifically, T2 will have accessible print and accessible weigh scales, examination tables, and cardiovascular equipment for participants.

Weakness:

None

General:

None
